# NGF/TRKA Promotes ADAM17-Dependent Cleavage of P75 in Ovarian Cells: Elucidating a Pro-Tumoral Mechanism

**DOI:** 10.3390/ijms23042124

**Published:** 2022-02-15

**Authors:** Maritza P. Garrido, Christopher Vallejos, Silvanna Girardi, Fernando Gabler, Alberto Selman, Fernanda López, Margarita Vega, Carmen Romero

**Affiliations:** 1 Laboratorio de Endocrinología y Biología de la Reproducción, Hospital Clínico Universidad de Chile, Santiago 8380456, Chile; mgarrido@hcuch.cl (M.P.G.); cvallejos@ispch.cl (C.V.); silvanna.girardi@bq.uchile.cl (S.G.); flopez@hcuch.cl (F.L.); mvega@hcuch.cl (M.V.); 2 Departamento de Obstetricia y Ginecología, Facultad de Medicina, Universidad de Chile, Santiago 8380453, Chile; selmanalberto@gmail.com; 3 Departamento de Patología, Escuela de Medicina, Hospital San Borja Arriarán, Universidad de Chile, Santiago 8360160, Chile; gablerf@uchile.cl; 4 Instituto Nacional del Cáncer, Santiago 8380455, Chile

**Keywords:** NGF, TRKA, P75, ADAM17, secretase, ovarian cancer, ovarian cells

## Abstract

Nerve growth factor (NGF) and its high-affinity receptor TRKA are overexpressed in epithelial ovarian cancer (EOC) displaying a crucial role in the disease progression. Otherwise, NGF interacts with its low-affinity receptor P75, activating pro-apoptotic pathways. In neurons, P75 could be cleaved by metalloproteinases (α and γ-secretases), leading to a decrease in P75 signaling. Therefore, this study aimed to evaluate whether the shedding of P75 occurs in EOC cells and whether NGF/TRKA could promote the cleavage of the P75 receptor. The immunodetection of the α-secretase, ADAM17, TRKA, P75, and P75 fragments was assessed by immunohisto/cytochemistry and Western blot in biopsies and ovarian cell lines. The TRKA and secretases’ inhibition was performed using specific inhibitors. The results show that P75 immunodetection decreased during EOC progression and was negatively correlated with the presence of TRKA in EOC biopsies. NGF/TRKA increases ADAM17 levels and the fragments of P75 in ovarian cells. This effect is abolished when cells are previously treated with ADAM17, γ-secretase, and TRKA inhibitors. These results indicate that NGF/TRKA promotes the shedding of P75, involving the activation of secretases such as ADAM17. Since ADAM17 has been proposed as a screening marker for early detection of EOC, our results contribute to understanding better the role of ADAM17 and NGF/TRKA in EOC pathogenesis, which includes the NGF/TRKA-mediated cleavage of P75.

## 1. Introduction

Epithelial ovarian cancer (EOC) is a neoplasm with unspecific symptomatology and late diagnosis, with a 5-year relative survival rate of around 31% when diagnosed in late stages [1,2,3]. Neurotrophins and their receptors display an important role in EOC progression. For instance, nerve growth factor (NGF) and its high-affinity receptor tropomyosin receptor kinase A (TRKA) are highly expressed in advanced stages of the disease [4]. They are involved in fundamental processes during EOC progression, such as cell proliferation, survival, migration, and angiogenesis [4,5,6,7,8,9]. NGF can exert its effects binding different neurotrophin receptors. It binds preferentially to the TRKA receptor, but it could also bind to the P75 neurotrophin receptor (P75), considered a low-affinity NGF receptor [10,11]. Previous reports have shown that TRKA is overexpressed in advanced stages of EOC and that its activation is involved in the progression of the disease [4,12]. However, the role of the P75 receptor in EOC is less known.

The P75 receptor is a transmembrane glycoprotein, a member of the tumor necrosis factor (TNF) receptor superfamily [13]. It has a conserved intracellular death domain [14]. The interaction of P75 with the co-receptor sortilin and the pro-domain of the neurotrophins activates cell death signaling [15,16], suppressing tumoral growth and inducing apoptosis in several epithelial cancer cells [17,18,19,20]. During the progression of prostate cancer, P75 expression is progressively lost [21] and the downregulation of P75 increases its tumorigenic potential [11]. In oligodendrocytes, NGF/TRKA activates pro-survival signals, which rescue these cells from apoptosis involving MAPK activation, but also simultaneous suppression of P75-dependent signaling [22], suggesting that NGF/TRKA could modulate P75 signaling. Because EOC cells express high levels of TRKA [4], it is rational to contemplate that TRKA-dependent regulation of P75 may be occurring in ovarian cells.

In PC12 cells, the activation of TRKA by NGF favors the activation of some metalloproteinases (secretases), increasing the proteolytic cleavage of P75 in both extra and intracellular domains [23]. Therefore, the intact P75 receptor (P75 full-length) can be cleaved, producing three fragments: (1) P75-extracellular domain (P75-ECD), which is released to the interstice; (2) P75-membrane-bound carboxy-terminal fragment (P75-CTF), which remains anchored to the cell membrane; and (3) P75-soluble intracellular domain (P75-ICD), which is released to the cell cytoplasm [24,25].

**Figure 1:**
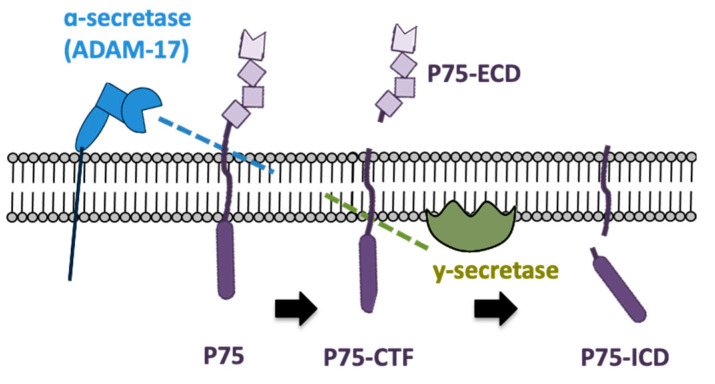
Fragments of the P75 receptor originated by α and γ-secretases. The P75 receptor could be shed by α-secretase (ADAM17), producing the fragment P75-extracellular domain (P75-ECD) and a P75-membrane-bound carboxy-terminal fragment (P75-CTF). This remaining fragment is now shed by γ-secretase, originating a P75-soluble intracellular domain (P75-ICD).

The shedding of the P75 receptor is sequential, beginning with an α-secretase, such as ADAM17, which generates the P75-CTF fragment (~27 kDa) [25]. It is known that γ-secretase aims at proteins that have already undergone cleavage, like P75-CTF, so γ-secretase cleaves this fragment, forming the P75-ICD fragment (~20 kDa) [24,25], as illustrated in Figure 1.

The α-secretase ADAM17 is strongly expressed in high-grade cancers and biopsies from patients with low survival rates, and thus is considered a marker of carcinogenesis progression and poor prognosis [26,27,28,29,30]. In blood samples from patients with EOC, a high concentration of ADAM17 is detected in the early stages of the disease [31], and in vitro experiments show that the inhibition of ADAM17 enhances cisplatin-induced apoptosis and decreases the cell viability of EOC cells [32].

In primary cultures of prostate adenocarcinoma, high levels of TRKA and low levels of P75 are detected [33]. Since NGF and its high-affinity receptor TRKA are overexpressed in EOC [4], and given that ADAM17 could be contributing to EOC progression, the current work aims to evaluate whether the cleavage of P75 occurs in ovarian tumors and EOC biopsies. Additionally, this study intends to evaluate the possible association between TRKA activation and the shedding of P75 by ADAM17 and γ-secretase in ovarian cells. Because ADAM17 has been proposed as a complementary marker to CA-125 for the early detection of EOC, and given that NGF/TRKA could modulate ADAM17, the current study contributes to a better understanding of the role of ADAM17 and NGF/TRKA in EOC pathogenesis.

## 2. Results

### 2.1. Detection of ADAM17 and TRKA in Epithelial Ovarian Cancer Biopsies and Ovarian Cells

**Figure 2:**
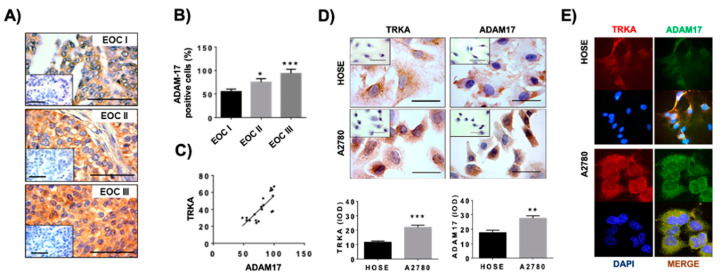
Presence of ADAM17 and TRKA in EOC biopsies and ovarian cell lines. (**A**): representative pictures of ADAM17 immunodetection in EOC tissues with different degrees of cell differentiation (EOC I: highly differentiated, EOC II: mildly differentiated, EOC III: poorly differentiated). Barr = 100 μm. N = 6 biopsies per group. (**B**): semiquantitative analysis of positive cells to ADAM17 in EOC tissues. * = *p* < 0.05 and *** = *p* < 0.01 vs. EOC I (Mann–Whitney test). (**C**): linear regression of TRKA and ADAM17 immunodetection in EOC tissues. Pearson coefficient = 0.7563 (*p* < 0.001). (**D**): TRKA and ADAM17 immunodetection in ovarian cell lines HOSE and A2780 and semiquantitative analysis using integrated optical density (IOD) of pictures. ** = *p* < 0.01 and *** = *p* < 0.001 (Mann–Whitney test). N = 3 independent experiments. Barr = 50 μm. (**E**): immunofluorescence of TRKA (red) and ADAM17 (green) in ovarian cell lines. Blue: cell nuclei fluorescence by addition of 4,6-diamidino-2-phenylindole (DAPI); yellow: merge of captures (TRKA and ADAM17 immunodetection). Results are expressed as the mean ± standard error of the mean (SEM).

In order to study the presence of ADAM17 in EOC tissues with different cell differentiation stages (EOC I: highly differentiated, EOC II: mildly differentiated, EOC III: poorly differentiated), the immunodetection of ADAM17 was performed in ovarian biopsies. Because we previously reported that the TRKA receptor increases during EOC progression [4], the same tissues were analyzed to assess ADAM17 immunodetection, using an antibody that recognizes the active form of this α-secretase. Results showed that ADAM17 was mainly found in epithelial ovarian cells (Figure 2A), with higher immunodetection in poorly differentiated EOC tissues (EOC III) (*p* < 0.01; Figure 2A,B). Besides, a positive correlation between ADAM17 and TRKA immunodetection was evidenced in the EOC biopsies (Figure 2C).

Otherwise, ADAM17 and TRKA immunodetection were assessed in two ovarian cell lines: HOSE and A2780 cells. The first are immortalized epithelial surface ovarian cells that did not display malignant features, unlike A2780 cells. Results show that immunodetection of both TRKA and ADAM17 was higher in A2780 cells compared to HOSE cells (*p* < 0.001 and *p* < 0.01, respectively; Figure 2D). In addition, these proteins display a cytoplasmic localization, and they are close to each other (Figure 2E).

### 2.2. Levels of P75 during EOC Progression

**Figure 3:**
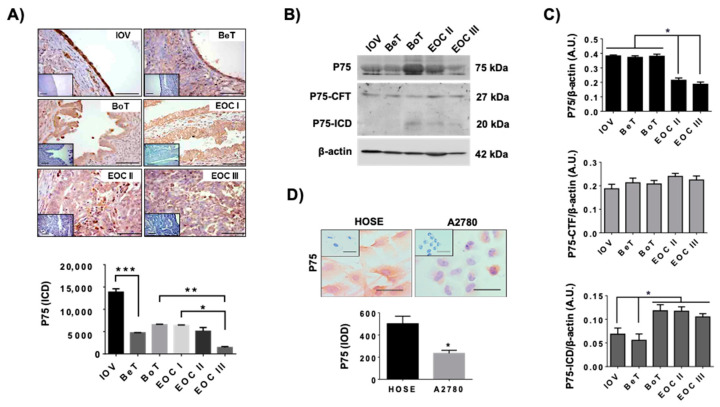
Levels of P75 and its fragments during EOC progression. (**A**): representative images and semi-quantification of P75 immunodetection (full-length receptor) in ovarian biopsies: IOV = inactive ovaries (from post-menopausal women). BeT = benign tumors (cystadenomas), BoT = borderline tumors, EOC = epithelial ovarian cancer; well-differentiated (EOC I), moderately differentiated (EOC II), and poorly differentiated (EOC III). * = *p* < 0.05, ** = *p* < 0.01 and *** = *p* < 0.001 as indicated (Kruskal–Wallis test and Dunn’s post-test). N = 6 biopsies per group. (**B**): representative image of Western blot of P75 (full-length receptor), P75 membrane-bound carboxy-terminal fragment (P75-CTF), and P75 intracellular domain (P75-ICD) during EOC progression. (**C**): semi-quantification of immunoblots of P75 and its fragments. N = 6 biopsies per group. * = *p* < 0.05 as indicated (Kruskal–Wallis test and Dunn’s post-test). (**D**): P75 immunodetection in ovarian cell lines and its semi quantification in 3 independent experiments. * = *p* < 0.05 (Mann–Whitney test). Barr = 50 μm. Results are expressed as the mean ± standard error of the mean (SEM).

To determine whether P75 levels change during EOC progression, immunodetection of this receptor was performed in inactive ovaries from postmenopausal women (IOV), benign and borderline tumors (BeT, BoT), and EOC tissues. As Figure 3A shows, the presence of P75 decreased during EOC progression, being higher in inactive ovaries compared to ovarian tumors and EOC (*p* < 0.001). Importantly, the lowest levels of P75 were detected in poorly differentiated EOC (EOC III; *p* < 0.001).

To evaluate if the shedding of P75 occurs in EOC biopsies, the intact receptor and its fragments were assessed in both benign and malignant ovarian tissues. As Figure 3B,C shows, the levels of P75 (full-length receptor) decreased in EOC samples (*p* < 0.05) compared to IOV or ovarian tumors (*p* < 0.05). By contrast, the levels of the P75-ICD fragment were higher in borderline tumors and EOC compared to inactive ovaries or benign tumors (*p* < 0.05; Figure 3C), which indicates that the shedding of the P75 receptor is occurring as EOC progresses to later stages. Additionally, as expected, A2780 cells (EOC cell line) showed lower levels of P75 (full-length receptor) compared to HOSE cells (*p* < 0.05; Figure 3D).

### 2.3. Levels of P75 and Its Fragments by NGF/TRKA in Human Ovarian Cell Lines

**Figure 4:**
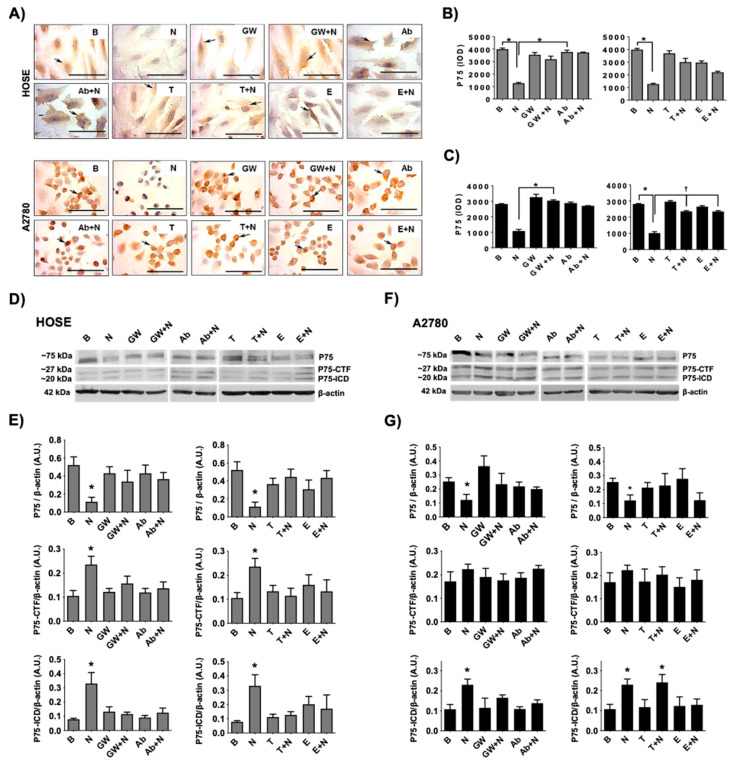
NGF/TRKA increases P75 cleavage in ovarian cells. Ovarian cells (HOSE and A2780) were stimulated with: NGF (N, 100 ng/mL) for 24 h and the following inhibitors (added 1 h after the addition of NGF): TRKA inhibitor GW441756 (GW, 20 nM), a neutralizing antibody against NGF (Ab, 5 μg/mL), ADAM17 inhibitor TAPI-0 (T, 10 μm), and the γ-secretase inhibitor Compound E (E, 200 mM). A condition without treatment (B, basal) was also included. (**A**): representative pictures of P75 immunodetection in ovarian cells under the different stimuli. (**B**,**C**): semi-quantification of P75 immunodetection using integrated optical density (IOD). (**D**): representative immunoblot of P75 fragments (P75-CTF and P75-ICD) using β-actin as a loading control. (**E**–**G**): densitometric semi-quantification of the immunoblot. N = 3 independent experiments. Barr = 100 μm. * = *p* < 0.05 with respect to the basal condition or as indicated (Kruskal–Wallis test and Dunn’s post-test). † = *p* < 0.05 as indicated according to the Mann–Whitney test. Results are expressed as the mean ± standard error of the mean (SEM).

To determine whether NGF/TRKA-dependent shedding of P75 occurs in ovarian cells, immunodetection of P75 and its fragments was performed in ovarian cells stimulated with NGF, the specific TRKA inhibitor GW441756 (GW), and a neutralizing antibody against-NGF (Ab). The results indicate that full-length receptor P75 decreased in ovarian cell lines stimulated with NGF (*p* < 0.05; Figure 4A,B). In addition, NGF increased the presence of P75 fragments (P75-CFT and P75-ICD) in both HOSE and A2780 cells (*p* < 0.05; Figure 4D–G). Importantly, these effects were prevented using the TRKA inhibitor or a neutralizing antibody against NGF (Figure 4D–G), indicating that NGF stimulation increases the cleavage of P75 in a TRKA-dependent manner.

To study whether ADAM17 and γ-secretase are involved in the NGF/TRKA-dependent shedding of P75, ovarian cells were treated with TAPI-0 (ADAM17 inhibitor, T) and the compound E (γ-secretase inhibitor, E). As shown in Figure 4B–G, the treatment with TAPI-0 prevented the NGF-mediated decrease in the P75 receptor in the ovarian cell lines (*p* < 0.05). In addition, the treatment with compound E prevented the NGF-dependent increase in the P75-ICD fragment in these cells (*p* < 0.05).

These results indicate that NGF/TRKA produces the shedding of P75 involving ADAM17 and γ-secretase activation in ovarian cells.

## 3. Discussion

Neurotrophins and their receptors are strongly involved in the progression of several kinds of cancers including EOC [4,7,34,35]. Both NGF and its high-affinity receptor TRKA are overexpressed in advanced stages of the disease [4] and the enhanced signaling of NGF/TRKA favors important processes such as cell proliferation, migration, and angiogenesis [4,5,9]. In neuronal cells, NGF/TRKA activates ADAM17, producing a shedding of the P75 receptor and the decrease in P75 canonical signaling [23,36]. In the current work, we showed that ADAM17 levels are correlated with TRKA levels in EOC biopsies and NGF/TRKA-dependent shedding of P75 is produced in ovarian cells, which could contribute to EOC progression. Undifferentiated EOC cells undergo an epithelial-to-mesenchymal transition, favoring metastasis and decreasing the patient’s survival [37]. Interestingly, we found a significant decrease in P75 levels (full-length receptor) in more advanced EOC tissues; meanwhile, ADAM 17 and P75-ICF reached the highest levels, suggesting that the shedding of P75 could be involved in the EOC dissemination.

P75 displays anti-tumoral effects in several in vitro models, such as prostate, bladder, and breast cancer cells [38,39,40]. The cleavage of P75 by ADAM17 inactivates its canonical signaling by the alteration of its ligand-binding site and the reduction of the sortilin/P75 complex formation, decreasing the signaling related to the programmed cell death [10,24]. This cleavage not only affects the canonical signal of P75 but also could activate other signaling pathways. For instance, the intracellular fragment of P75 (P75-ICD), produced from the sequential cleavage of P75 by ADAM17 and γ-secretase, acts as a scaffolding protein that favors the cell signaling in a ligand-independent manner [25]. Experiments performed in HEK293 and HeLa cells that overexpress the fragment P75-ICD evidenced a nuclear accumulation of this fragment in nuclei, and its binding with the cyclin E1 promoter upon NGF stimulation [41], suggesting that P75-ICD could regulate the transcription of specific genes.

**Figure 5:**
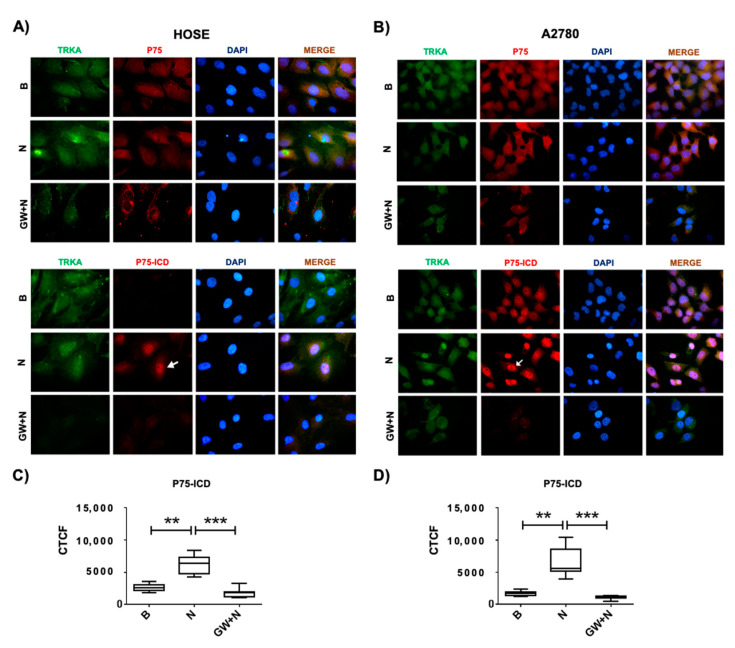
Cellular localization of P75 by NGF effect in EOC cell lines. Ovarian cells were treated with NGF (100 ng/mL, 24 h), NGF plus the specific TRKA inhibitor GW441756 (GW, 20 nM, added 2 h before NGF) or without treatment (B, basal condition). (**A**,**B**): immunodetection of TRKA, P75, and P75 intracellular domain (P75-ICD) in HOSE and A2780 cells, respectively. (**C**,**D**): semi-quantitative analysis of immunofluorescence of P75-ICD in HOSE and A2780 cells, respectively. Nuclear detection of P75-ICD is indicated with white arrows. Relative fluorescence was expressed as corrected total cell fluorescence (CTCF). ** = *p* < 0.01 and *** = *p* < 0.01 (Kruskal–Wallis test). Results are presented as a boxplot with whiskers from minimum to maximum.

In agreement with these results, studies have shown that both TRKA and P75 are expressed in advanced stages of ovarian carcinoma [4,42]. P75 expression is elevated in effusions while TRKA shows an opposite trend [42]. This supports the idea that TRKA induces the shedding of P75 receptor in EOC cells, and the fragments released as a result of P75 cleavage, could be detected in ovarian cancer effusions [42]. Results shown in Figure 5 lean toward this theory, because after NGF stimulation, the P75-ICD fragment could be visualized in a perinuclear localization, especially in A2780 cells, and this effect is prevented in the presence of a specific TRKA inhibitor (GW).

In agreement with these results, researchers have shown that the generation of the P75-ICD fragment enhances trkA signaling in neurons, causing conformational changes within the extracellular domain of trkA and enhancing the neurotrophic signaling [43,44]. Similarly, P75 facilitates the activation of trkB by BDNF promoting survival pathways in rat hippocampal neurons [45]. These findings suggest that the shedding of P75 not only decreases its pro-apoptotic signaling but also favors neurotropic signaling, which has been associated with cancer progression.

Contrary to our results, some researchers have shown that P75 could promote cell migration and invasion, thus contributing to cancer resistance [46,47]. On the one hand, a decrease in P75 in tumoral tissues compared to non-tumoral tissues has been described [48,49], but on the other hand, some authors have reported a higher expression of P75 in primary tumors compared to effusions from patients with breast carcinoma [50]. These discrepancies could be explained at least in part by the use of antibodies that recognize different aminoacidic sequences of P75 (and then the intact receptor or its fragments), and also by the interaction of P75-ICD with other receptors producing its trans-activation (for instance, TRK receptors), which could yield P75-mediated tumoral effects.

Because ADAM17 displays an important role in inflammatory diseases, it has been proposed as a therapeutic target in several kinds of cancer [51], including EOC [32,52,53]. This knowledge has motivated the development of pharmacologic inhibitors and antibodies targeting ADAM17 as anti-tumoral therapy, which have been tested in some preclinical models of EOC [51,52,53].

Recent work has shown that serum ADAM17 could be a promising screening marker for EOC [31,51]. CA125 is a serum marker widely used to evaluate response to treatment in ovarian cancer patients. However, its accuracy in population-based screening is still poor (its use is not recommended in asymptomatic women at risk) [54]. In addition, CA125 levels increase in benign diseases such as menstruation, pregnancy, benign pelvic tumors, pelvic inflammatory diseases, ovarian hyperstimulation syndrome, peritonitis, endometriosis, and others [55], which makes its adequate interpretation difficult. Since higher ADAM17 concentrations have been detected in patients with EOC in the early stages [31], the combined use of CA125 and ADAM-17 could be used to detect ovarian carcinoma with better sensibility and specificity. This should be tested in future research.

**Figure 6:**
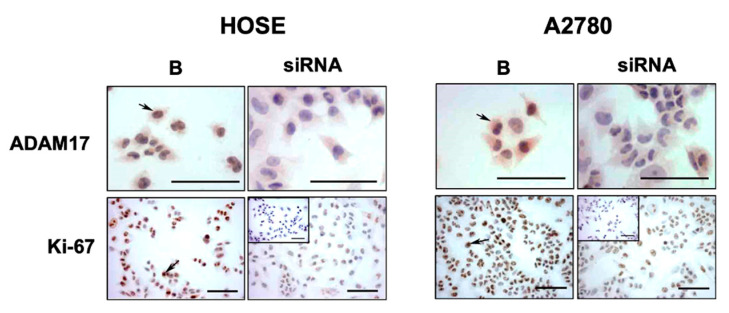
Downregulation of ADAM17 decreases Ki-67 immunodetection in ovarian cells. Ovarian cell lines (HOSE and A2780) were transfected with a siRNA targeting ADAM17. Cells were fixed and immunocytochemistry to detect Ki-67 (brown stain) was performed according to the methodology section. Barr = 100 μm. Examples of positive cells are pointed by arrows.

To validate the importance of ADAM17 as a tumor promoter in our in vitro models, we performed an experiment using a small interference RNA (siRNA) against ADAM17 (Figure 6). At first glance, the downregulation of ADAM17 produced an evident decrease in Ki-67 immunodetection. Based on this, and considering that P75-ICD binds cyclin E1 promoter [41] (which is required for cell cycle G1/S transition [56]), it is reasonable to think that downregulation of ADAM17 prevents the proteolytic cleavage of P75 (by itself and by γ-secretase). This could yield a decrease in the generation of P75-ICD, impairing the growth potential of ovarian cells.

**Figure 7:**
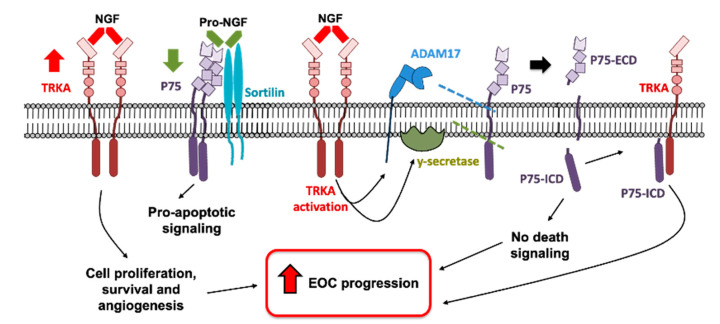
Possible mechanisms by which NGF/TRKA increases the shedding of P75, promoting EOC progression. In EOC cells, TRKA is overexpressed (which increases cell proliferation and survival signaling) and P75 is downregulated (decreasing pro-apoptotic signaling). NGF/TRKA activates ADAM17 producing the shedding of P75 and generating the P75 membrane-bound carboxy-terminal fragment (P75-CTF) and P75-extracellular domain (P75-ECD). Hence, y-secretase performs the second shedding to P75, which generates a P75 soluble intracellular domain (P75-ICD) fragment. These results suggest a decrease in pro-apoptotic signaling and an enhanced TRKA signaling, which contribute to EOC progression.

In summary, this preliminary report indicates that, in EOC tissues, P75 levels decreased during the disease progression. In ovarian cells, NGF/TRKA produced a decrease in P75-full length and increased P75 fragments involving the activation of ADAM17 and γ-secretase. Therefore, the decrease in P75 in advanced stages of EOC cells could be mediated at least in part by TRKA-dependent shedding. In this context, the results suggest that the shedding of P75 could promote cancer progression by a decrease in P75 signaling and the generation of P75 fragments (P75-CTF and P75-ICD) which may enhance TRK signaling in ovarian cells (Figure 7). However, the role of P75 fragments in tumorigenesis is still unknown and should be further studied in the future.

## 4. Materials and Methods

### 4.1. Tissue Samples

A total of 36 patients were recruited at the Clinical Hospital of the University of Chile and the National Institute of Cancer. Each patient signed an informed consent approved by the Ethics Committee of both institutions. Tissues were classified by an expert pathologist in benign tumors (Ben-T, n = 6), borderline tumors (Bord-T, n = 6), and serous epithelial ovarian cancer (EOC, n = 18) which in turn were classified into well-differentiated (EOC I), moderately differentiated (EOC II), and poorly differentiated (EOC III). Additionally, a group with samples of inactive ovaries (IOV, n = 6) from post-menopausal women undergoing hysterectomy with oophorectomy without ovarian pathologies were included. All patients were menopausal or postmenopausal at the time of the study, without statistical differences in age. The median age of the volunteers was 61 years (with a 95% of confidence interval 50–66 years).

Ovarian tissue samples were divided into two. One piece of each sample was frozen in liquid nitrogen and maintained at −80 °C for Western blotting; the other piece was embedded in paraffin for immunohistochemistry.

### 4.2. Cell Culture and Treatments

Two ovarian cell lines were used to perform experimental assays: HOSE and A2780. HOSE cells are human ovarian surface epithelial cells from a postmenopausal woman which are immortalized with SV40-Tag [57]. A2780 is a human ovarian cancer cell line with epithelial morphology that was established using tumor tissue from an untreated patient [58]. These cell lines are representative models of non-cancerous ovarian epithelium and EOC, respectively [59,60,61]. Ovarian cells were cultured in DMEM/Ham-F12 medium without phenol red (Sigma-Aldrich Co. Saint Louis, MO, USA) supplemented with 10% FBS in the presence of 100 U/mL penicillin G, 100 μg/mL streptomycin sulfate, and 25 g/mL amphotericin B (Hyclone™, Thermo Fisher Scientific, Waltham, MA, USA) and cultured at 37 °C with 5% CO_2_.

For Western blot experiments, 700,000 cells/plate were cultured in 6-well plates. For immunocytochemistry, 200,000 cells/plate were cultured in 4-well Lab-Tek^®^ II Chamber Slides™ (Waltham, MA, USA). Cells were serum-deprived for 24 h and then were stimulated with NGF (100 ng/mL, Sigma-Aldrich, St. Louis, MO, USA) for 24 h. In addition, cells were treated with TRKA specific inhibitor GW441756 (GW, 20 nM, #2238, Tocris, Minneapolis, MN, USA), ADAM17 inhibitor TAPI-0 (T, 10 µM, sc-203410, Santa Cruz Biotechnology, Dallas, TX, USA), γ-secretase inhibitor Compound E (E, 200 mM, #D00137679, Calbiochem, San Diego, CA, USA), or an anti-NGF neutralizing antibody (Ab 5 μg/mL; Abcam 6199, Cambridge, UK). The inhibitors were added 1 h before NGF. Transfection of ADAM17 siRNA was performed using on-TARGET plus ADAM17 siRNA (#J-003453-06, Thermo Fisher Scientific, Waltham, MA, USA) according to the manufacturer’s instructions.

### 4.3. Antibodies

The antibodies used for immunohistochemistry, immunocytochemistry, and/or Western blot were: mouse monoclonal anti-β-actin (A 5441, Sigma-Aldrich); mouse monoclonal anti-ADAM17 (ab57484, Abcam, Cambridge, UK), rabbit polyclonal anti-P75 intracellular domain (#07-476 Millipore, Temecula, CA, USA), mouse monoclonal anti-P75 that recognizes extracellular domain (N 5408 Sigma-Aldrich), rabbit polyclonal anti-Ki-67 antibody (#AB9260 Millipore), anti-rabbit IgG secondary antibody (#074-1516, KPL, Gaithersburg, MD, USA), and anti-mouse IgG secondary antibody (#074-1806, KPL).

### 4.4. Western Blotting

In brief, fresh tissue specimens and cell samples were homogenized and lysed on ice using a cell RIPA lysis with protease and phosphatase inhibitors (Thermo Fisher Scientific). After centrifugation at 10,000× *g* for 20 min at 4 °C, protein concentrations were determined using the BCA protein assay kit (Pierce, IL, USA). Total proteins (40 μg) were separated in a 12% SDS-PAGE gel and transferred to a nitrocellulose membrane. Blots were blocked for 1 h in tris-buffered saline with Tween 20 (TBST) containing 5% nonfat dry milk. Subsequently, the blots were washed three times for 5 min each in TBST and then incubated overnight at 4 °C with antibodies against P75 (1:500) and P75-ICD (1:100). β-actin antibody (1:20,000) was incubated for 1 h at room temperature. The blots were washed three times for 5 min each with TBST, followed by incubation while rocking for 1 h at room temperature with anti-rabbit IgG or anti-mouse IgG conjugated to peroxidase antibody (1:5000). After washing three times for 10 min with TBST, the bound antibodies were detected with an enhanced chemiluminescence system using Syn Gene G: box Chemi-XT4 (Gene Sys, Cambridge, UK). Band intensities were quantified by scanning densitometry utilizing the UN-SCAN-IT software, version 6.1. The results were expressed as a ratio with β-actin values.

### 4.5. Immunohistochemistry

Immunostaining was performed on 5-μm sections of formalin-fixed paraffin-embedded ovarian biopsies. Briefly, tissue sections were deparaffinized in xylene and hydrated in a series of graded alcohols. The sections were reconstituted with 10 mM of sodium citrate buffer at 95 °C for 20 min. Endogenous peroxidase activity was prevented by incubating the samples in 3% hydrogen peroxide for 5 min. Nonspecific antibody binding was blocked with kit Histostain SP (Zymed Laboratories Inc., San Francisco, CA, USA). The samples were incubated for 18 h at 4 °C with the primary antibodies (1:1000 for anti-P75 and 1:1000 for anti-ADAM17)). Negative controls were analyzed on adjacent sections incubated without the primary antibody and using non-immune species-specific antisera. The secondary antibody (1:300) was incubated at 37 °C for 30 min and 3,3’-diaminobenzidine (DAB) (#K3467, Dako, Carpinteria, CA, USA) was used to visualize peroxidase activity. Counterstaining was carried out with hematoxylin. The material was dehydrated and cleared in xylene, mounted in coverslips with Entellan new^®^ (#107961, Merck Millipore Corporation, Billerica, MA, USA), and examined under an optical microscope. A total of 10 captures of each sample were taken and the results were averaged. These photos were obtained from superior, central, and inferior quadrants of the sample previously recognized and marked by an expert pathologist. Images were analyzed using Image Pro Plus 6.1 software and integrated optical density (IOD) was obtained and expressed as arbitrary units (AU).

### 4.6. Immunocytochemistry

Cells were fixed in 4% paraformaldehyde in PBS pH 7.4 for 15 min at room temperature and permeabilized with 0.1% Triton X100 in PBS for 10 min at room temperature. Endogenous peroxidase blocking was performed with 3% hydrogen peroxide for 15 min. Non-specific binding was blocked using 5% milk in PBS for 10 min. After incubation overnight with anti-P75 (1:1000) or anti-Ki67 (1:100), the anti-rabbit secondary antibody (1:300) was applied for 30 min at 37 °C. Positive cells were visualized using a kit with DAB. Samples were counterstained with Harris hematoxylin (1:5), dehydrated, and mounted to be visualized in an optical microscope.

Immunofluorescence was performed in the same conditions as immunocytochemistry, but after fixation, cells were stained and mounted with Prolongue™ 4′,6-diamidino-2-phenylindole (Dapi) (Thermo Fisher Scientific) and stored to 4 °C after microscope examination.

Images were acquired with a MicroPublisher 3.3 RTV camera (Q Imaging, Surrey, BC, Canada) (Tapia et al. 2011). The evaluation was done by Image Pro Plus 6.1 software, measuring IOD expressed as arbitrary units (AU).

### 4.7. Statistical Evaluation

Data were evaluated using nonparametric tests (Kruskal–Wallis and Dunn post-test or Mann–Whitney test) using the software Graph Pad Prism 5.2. *p*-values < 0.05 were considered significant.

## Data Availability

The data presented in this study are available on request from the corresponding author.
